# Trapping photons on the line: controllable dynamics of a quantum walk

**DOI:** 10.1038/srep04825

**Published:** 2014-04-28

**Authors:** Peng Xue, Hao Qin, Bao Tang

**Affiliations:** 1 Department of Physics, Southeast University, Nanjing, Jiangsu 211189, China; 2 State Key Laboratory of Precision Spectroscopy, East China Normal University, Shanghai 200062, China

## Abstract

Optical interferometers comprising birefringent-crystal beam displacers, wave plates, and phase shifters serve as stable devices for simulating quantum information processes such as heralded coined quantum walks. Quantum walks are important for quantum algorithms, universal quantum computing circuits, quantum transport in complex systems, and demonstrating intriguing nonlinear dynamical quantum phenomena. We introduce fully controllable polarization-independent phase shifters in optical pathes in order to realize site-dependent phase defects. The effectiveness of our interferometer is demonstrated through realizing single-photon quantum-walk dynamics in one dimension. By applying site-dependent phase defects, the translational symmetry of an ideal standard quantum walk is broken resulting in localization effect in a quantum walk architecture. The walk is realized for different site-dependent phase defects and coin settings, indicating the strength of localization signature depends on the level of phase due to site-dependent phase defects and coin settings and opening the way for the implementation of a quantum-walk-based algorithm.

Quantum walks (QWs)^1^,^2^,^3^ are the quantum mechanical analog of classical random walks (RWs), and hence can be used to develop quantum algorithms^4^,^5^,^6^,^7^, emerge as an alternative to the standard circuit model for quantum computing^8^,^9^,^10^, and represent one of the most promising resources for the simulation of physical system and important phenomena such as energy transport in photosynthesis^11^,^12^, quantum chaos^13^,^14^,^15^,^16^, Anderson localization^17^,^18^,^19^,^20^,^21^,^22^,^23^, and topological phases^24^.

A standard model of a one-dimension (1D) discrete-time QW consists of a quantum walker carrying a quantum coin. The walker goes back and forth along a line and the direction at each step depends on the result of a coin flip, which can be implemented by an arbitrary unitary operation in SU(2) following by a conditional position shift operation. The position variance of the walker *σ*^2^ = 〈*x*^2^〉 − 〈*x*〉^2^ is linear on the number of the steps for RWs and quadratic for QWs. The position distribution of a standard QW *P*(*x*) shows a ballistic diffusion and that of RWs diffusive spreading. Furthermore if the static disorder is introduced in the dynamics of QWs, by changing the interference pattern localization effect can be observed in a QW architecture—spreading more slowly than RWs^19^.

Experimental QWs began in 1999 and were performed with the frequency space of an optical resonator^2^. Several alternative realizations were quickly afterwards based on energy levels in nuclear magnetic resonance^25^, phase and position space of trapped ions^26^,^27^,^28^ and trapped neutral atoms^29^, photons in beam splitter array, in fiber loop and in waveguide structures^3^,^16^,^30^,^31^,^32^,^33^,^34^,^35^,^36^.

In this work, we report on the implementation of a discrete QW with site-dependent single-point phase defects (SPPDs), and implement methods suggested by Wójcik et al^19^. We investigate the evolution of single-photons moving in a discrete environment presenting SPPDs, casting light on the natural feature of the physical description of QWs such as general properties of diffusion modified by quantum or interference effects. Compared to the previous optical experiments on standard QWs (e.g. without static disorder)^3^,^32^,^33^,^34^,^35^,^36^, we use phase shifters (PSs) for realization of the site-dependent phases acquired by the walker at the certain position. Compared to the experimental realization of QWs with time-dependent phase defects^23^, our experiment on QWs with site-dependent phases is more close to nature and can be used to investigate localization effect on low-dimensional structure, which would be interesting in research on properties of low-dimensional materials.

## Results

The QW eliminates random evolution by trading the coin for a quantum two-level system, which, in our case, is the polarization state of a single photon: horizontal (H) and vertical (V). Furthermore the QW employs unitary dynamics by which the walker's position is entangled with the coin state.

We use the array of beam displacers (BDs) as interferometer network similar to the setup in the previous work^16^. By taking advantage of the intrinsically stable interferometers, our approach is robust and able to control both coin and walker at each step. Benefiting from the fully controllable implementation, we experimentally study the impact of the SPPD and coin bias on the localization effect in a QW architecture and the experimental results agree with the theoretical predictions. Compared to the previous experimental results which only simulated localization effect by trapping the walker in the original position *x* = 0, we experimentally localize the single-photons in different positions.

In our experiment, we are able to achieve 10 QW-steps, surpassing the previous limit of 7 QW-steps in a similar interferometric setup^3^. The challenge of our experiment is to realize specific polarizing-independent phase on each site via microscope slides with precise effective thickness as PSs and to keep high interference visibility for each step even with phase defect. By introducing controllable PSs in paths of the interferometers, We have managed to create these versatile interferometer networks which can be used in many other fields.

The coin state is encoded in the polarization |*H*〉 and |*V*〉 of the input single-photon which are generated by type-I spontaneous parametric downconversion (SPDC). In the basis {|*H*〉, |*V*〉}, the coin operation for each step is given by 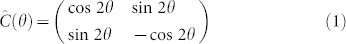
with *θ* ∈ (0°, 45°), consisting of a polarization rotation, which is realized with a half-wave plate (HWP) setting. For example, the Hadamard operator is realized with a HWP set to *θ* = 22.5°.

The walker's positions are represented by longitudinal spatial modes which are implemented by birefringent calcite BDs. The optical axis of each BD is cut so that vertically polarized light is directly transmitted and horizontal light undergoes a lateral displacement into a neighboring mode which interferes with the vertical light in the same mode. Each pair of BDs forms an interferometer.

The unitary operator for each step of a QW with a SPPD 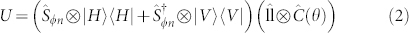
with the position shift operator 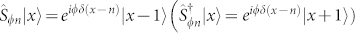 on the modes manipulates the wavepacket to propagate according to the polarization of the photons. Here *δ*(*x*) is the Dirac delta function which satisfies *δ*(0) = 1 and *δ*(*x*) = 0 for *x* ≠ 0. The translational symmetry of an ideal standard QW without SPPD is now broken by modifying the phase of the walker on each site, which can be realized by simply introducing PSs in the specific interferometer arms. PSs are placed in the certain modes *x* = *n*. By adjusting the relative angle between the PS and the following BD the effective thickness of the PS changes and the specific phase *ϕ* can be realized.

**Figure 1:**
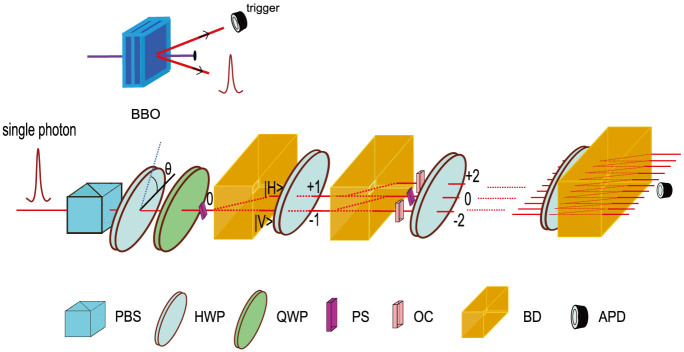
Experimental scheme for 10-step QW with site-dependent phase function. Single-photons created via type-I SPDC are injected to the optical network. Arbitrary initial coin states are prepared by a polarizing beam splitter (PBS), half-wave plates (HWPs) and quarter-wave plates (QWPs). Phase shifters(PSs) are placed in the corresponding spatial modes and the optical compensators (OCs) are used to compensate the temporal delay caused by PSs. Coincident detection of photons at avalanche photo-diodes (APDs, 7 ns time window) predicts a successful run of the QW.

**Figure 2:**
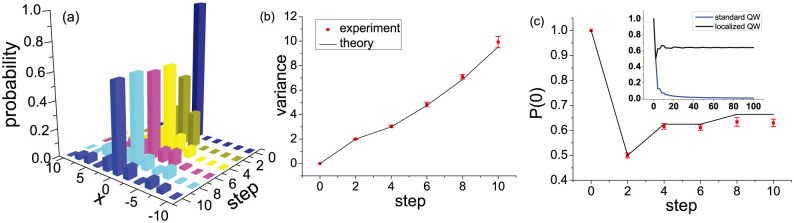
Localization effect in a QW architecture. (a) Experimental position distributions for successive steps of the Hadamard (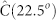) QW with the SPPD *ϕ* = 180*^o^* in the original position *x* = 0 and antisymmetric initial coin state up to 10 steps. (b) Measured position variance and (c) recurrence probability of localized QW for 1 to 10 steps, compared to theoretical predictions (solid lines). Error bars indicate the statistical uncertainty. Inset shows comparison of theoretical prediction of the recurrence probability of localized Hadamard QW with the SPPD *ϕ* = 180*^o^* and standard QW with *ϕ* = 0.

The first 10 steps of the QW with SPPD *ϕ* applied in the original position *x* = 0 are shown in Fig. 1 in detailed. The longitudinal spatial modes after the 1st step are recombined interferometrically at the 2nd step. In our experiment, we attain interference visibility of 0.998 (extinction ratio 1000:1) for each step, i.e., for each pair of sequential beam displacers. The photons emerge in the *N* + 1 spatial modes at the output of the *N*th step and are subsequently detected by avalanche photodiodes (APDs). The probabilities are obtained by normalizing photon counts on each site to total number of photon counts for the respective step. The measured probability distributions for 1 to 10 steps of a Hadamard QW with SPPD *ϕ* = 180° and the antisymmetric initial coin state 
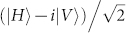
 are shown in Fig. 2a. An average distance 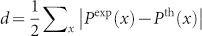 is 0.046 ensuring a good agreement between the measured probabilities and theoretic predictions after 10 steps. The walker state after 4 steps clearly shows the characteristic shape of a localization distribution: a pronounced peak of the probability 0.615 ± 0.011 in the original position *x* = 0 and the low probabilities in the side positions. In contrast to the ideal standard Hadamard QW the expansion of the wavepacket is highly suppressed and the probability of the walker returning to the original position is enhanced strictly and displays the signature of the localization effect.

In Figs. 2b and 2c, the position variance and recurrence probability, i.e., the probability of the walker returning to the original position, show the spread of the localized QW is much slower than that of the standard QW and the walker is trapped in the original position with high probability after the 4th step. While in the case of the 10-step standard Hadamard QW without SPPD the variance is given by 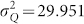, a lower variance occurs in the RW case with 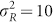. Our measured value *σ*^2^ = 9.586 ± 0.463 agrees well with the theoretical prediction 9.547 and shows an even slower spread than RW. The presented error bars include only statistical errors, calculated from the standard deviations of the values calculated by the Monte Carlo method. The measured recurrence probability exhibits the localization effect of QW with SPPD after the 4th step. Compared to the walker in a standard QW with SPPD showing a ballistic behavior, it is always trapped in the position *x* = 0 with high probability about 0.64 after 4 steps shown in Fig. 2c.

**Figure 3:**
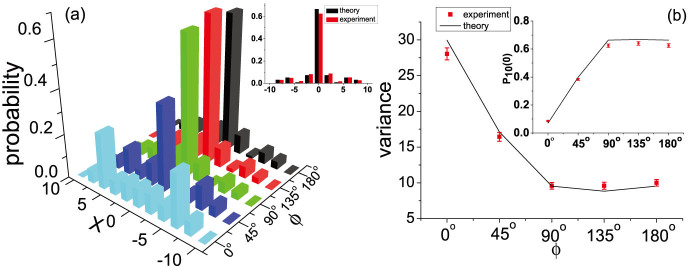
The strength of localization signature due to the level of phase defects. (a) Experimental data of probability distributions of the 10-step Hadamard QW with antisymmetric initial coin state and various single-point phase *ϕ* in the original position *x* = 0. The inset shows the probability distribution of the 10th-step Hadamard QW with SPPD *ϕ* = 135*^o^*. Red and black bars show experimental data and theoretical predictions respectively. (b) Measured position variance of the 10-step Hadamard QW v.s. *ϕ*, compared to theoretical predictions (solid lines). The inset shows the measured recurrence probabilities *P*_10_(0) after the 10 steps as a function of *ϕ*.

Our second experimental result highlights the full control of the implementation of the QW. In Fig. 3, we show the impact of single-point phase *ϕ* ∈ [0°, 180°] on the localization effect. In this case, we change the effective thickness of the PSs to realize different SPPDs in the original position. Fig. 3a shows the position distribution of the 10-step Hadamard QW changes as a function of the single-point phase *ϕ*. At the 10th step the recurrence probability *P*_10_(0) and the position variance *σ*^2^ as functions of *ϕ* are shown in Fig. 3b. For the antisymmetric initial coin state, the recurrence probability *P*_10_(0) increases with *ϕ* in the range [0°, 135°] and decreases in the range (135°, 180°]. The maximal probability is achieved (measured as 0.660 ± 0.012 and numerical simulated as 0.667) with *ϕ* = 135°. The localization effect occurs in the range [45°, 180°], which agrees with the analytic result. Thus with fixed coin toss and initial state, whether or not the localization effect can be observed depends on the choices of single-point phase applied in the original position.

**Figure 4:**
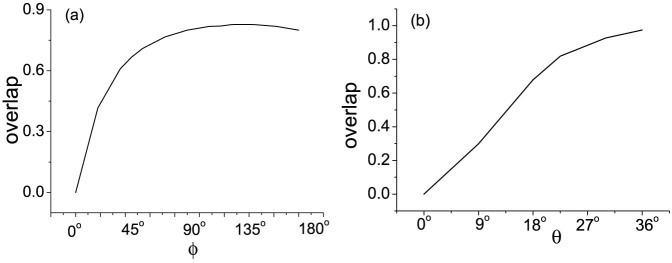
Explanation of localization effect in a QW architecture. (a) Overlap between the localized eigenstates of the unitary operation *U* of the Hadamard QW with SPPD in *x* = 0 and the initial state of the walker + coin system as a function of the phase *ϕ*. (b) Overlap between the localized eigenstates of *U* of the QW with SPPD *ϕ* = 180*^o^* in *x* = 0 and the initial state as a function of the coin bias *θ*.

The dependence of the localization effect on the phase *ϕ* can be explained^19^ by the overlap between the localized eigenstates of the single-step unitary operator *U* and the initial state of the walker + coin system shown in Fig. 4a. The eigenstates and the corresponding eigenvalues can be found by solving a set of recurrence equations generated by the unitary operator *U*. The number of the localized eigenstates of *U* depends on *ϕ*. In the range *ϕ* ∈ (0°, 45°) and *ϕ* ∈ (135°, 180°) there are two localized eigenstates. Whereas, in the range *ϕ* ∈ (45°, 135°) there are four such states. With the initial state 
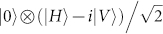
, the overlap increases from 0 to 0.828 with *ϕ* ∈ (0°, 135°) and decrease to 0.8 with *ϕ* ∈ (135°, 180°). The localization effect occurs in the range *ϕ* ∈ [45°, 180°] and becomes most notable with *ϕ* = 135°.

**Figure 5:**
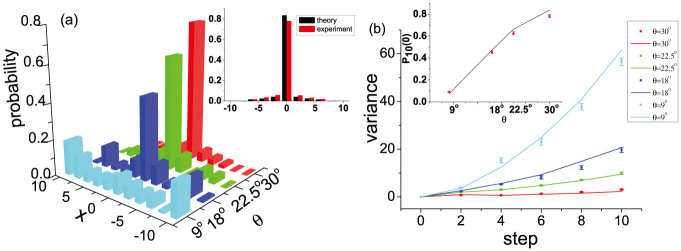
Influence on the localization effect in a QW architecture due to different coin settings. (a) Experimental data of probability distributions of the 10-step QW with SPPD *ϕ* = 180*^o^* in the original position *x* = 0, antisymmetric initial coin state and various coin bias *θ*. The inset shows the probability distribution of the 10th-step QW with the coin bias *θ* = 30*^o^*. Red and black bars show experimental data and theoretical predictions respectively. (b) Measured position variance of the localized QW with antisymmetric initial coin state for 1 to 10 steps, with respective theoretical simulation (solid lines). The inset shows the measured probabilities of the walker returning to the original position *P*_10_(0) at the 10th step with the antisymmetric initial coin state as a function of the coin bias *θ*. Some of the statistical error bars are smaller than the symbol size.

The next experimental result shown in Fig. 5 highlights the flexibility of our implementation with respect to the easy adjustability of the coin bias. Now we study the impact of the different coin biases on the localized QWs. Figure 5a shows the measured position distributions for the 10-step localized QW with antisymmetric initial coin state, SPPD *ϕ* = 180° in the original position *x* = 0 and different coin biases *θ* = 9°, 18°, 22.5°, 30° realized via different HWP settings. The walker is trapped in the original position with the proper choice of *ϕ*, which can also be observed from the position variances and recurrence probabilities in Fig. 5b. With the angle of HWP *θ* increasing, the mode of behaviour of the walker + coin system is transmitted from the diabatic (diabatic transition probability *D* = cos^2^ 2*θ* ~ 1) to adiabatic limit (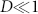). Harmin predicted that suppression of diffusion occurs in the form of almost perfect recurrences of the initial level population in the adiabatic limit, while in the diabatic limit, the recurrence will vanish^37^. Our experiment result agrees with the theoretic predictions and shows that with the HWP angle of *θ* = 9° and *D* = 0.905, in the diabatic limit the walker spreads widely and no recurrence occurs. Whereas, with the HWP angle increasing to *θ* = 30° (*D* = 0.095), the mode of the behaviour of the system is transmitted to the adiabatic limit, the diffusion is more suppressed and the recurrence probability increases. Thus the localization effect becomes more obvious in the adiabatic limit. This result can also be explained by the dependence of the overlap between the localized eigenstates and the initial state of the walker + coin system on the coin bias shown in Fig. 4b. With *θ* increasing from 0° to 30°, the overlap increases monotonically from 0 to 0.974, which suggests the localization effect becomes more notable.

**Figure 6:**
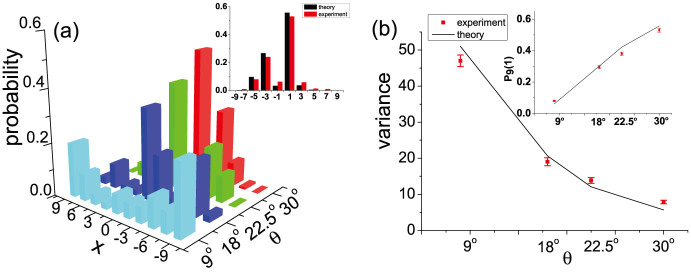
A 9-step QW with SPPD at *x* = 1. (a) Experimental data of probability distributions of the 9-step QW with antisymmetric initial coin state, SPPD *ϕ* = 180*^o^* in the position *x* = 1 and various coin bias *θ*. The inset shows the probability distribution of the 9th-step QW with the coin flipping 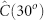. Red and black bars show experimental data and theoretical predictions respectively. (b) Measured position variance of the 9-step QW v.s. the coin bias *θ*, with respect to the theoretical predictions (solid line). The inset shows the measured probabilities of the walker returning to the *x* = 1 position *P*_9_(1).

Compared to the previous experimental results which only simulated Anderson localization of the QW by trapping the walker in the original position, we experimentally localize the single-photons in different positions, for example in *x* = 1. By inserting the PSs with proper effective thickness in the spatial mode *x* = 1 at each odd step, we can realize a localized QW with SPPD *ϕ* = 180° in *x* = 1. In Fig. 6, the walker appears in *x* = 1 with highest probability after 9 steps. With different coin settings, the localization effect becomes more notable when the system is transmitted from diabatic to adiabatic limit. The tendency of the position variance and the probability of the walker back to *x* = 1 depends on the coin bias *θ* in the same manner compared to the case of the localization in the original position.

The performance of our setup is limited only by imperfections of the optical components such as nonplanar optical surfaces and the coherence length of single photons, resulting in errors and decoherence. The most significant source of systematic errors in our setup is the imperfect coherence visibility of the BD interferometer. A limitation for the maximal step number is given by the size of the clear aperture of BDs. For example, for 10-step QW, the effective diameter of the clear aperture of the BD with beam separation 3 mm needs to be larger than 30 mm. However this problem is not intrinsic to this implementation, since the BDs with large enough clear aperture and strictly planar surface can realize the large-step QW.

## Conclusion

In summary, we implement a stable and efficient way to realize QWs embedded in a broader framework and show the phase defects can influence the evolution of wavepackets. The QW with SPPD has the single-photons localized in the certain position. Our experiment benefits from the high stability and full control of both coin and walker at each step and in each given position. The versatility of our setup allows for extensions, such as the realization of multi-particle QWs, in which richer choices of coin flipping and defects would help us to study the topology of arbitrary graphs and develop the applications such as quantum state transfer and energy transportation problems. The localization can also be used to filter and to trap particles, which would find applications in quantum algorithms and quantum state engineering. Our results show a new realm of QW phenomena and our new interferometer phase-shift control provides a valuable new tool for exploring QWs with various potentials. Furthermore, the versatile interferometer network with high interference visibility can be used in many other fields of optical quantum information processing.

## Methods

### Photon-pair generation

The non-degenerated polarization degenerate photon pairs generated via type-I SPDC in two 0.5 mm-thick nonlinear-*β*-barium-borate (BBO) crystals cut at 29.41*^o^*, pumped by a 400.8 nm CW diode laser (LBX-405-100, Oxxius) with up to 100 mW of power. For 1D QWs, by triggering on one photon, the other at wavelength 800 nm is prepared into a single-photon state. A polarizing beam splitter (PBS) following by waveplates allow generation of any polarized state of single photon (e.g. any initial coin state). Interference filters determine the photon bandwidth 3 nm and then individual downconverted photons are steered into the optical modes of the linear-optical network formed by a series of birefringent calcite BDs, HWPs and PSs. Output photons are detected using APDs (SPCM-AQRH-14-FC) with dark count rate of <100 s^−1^ whose coincident signals—monitored using a commercially available counting logic (id800-TDC)—are used to post-select two single-photon events. The total coincident counts are about 300 s^−1^ (the coincident counts are collected over 60 s). The probability of creating more than one photon pair is less than 10^−4^ and can be neglected.

### Beam displacer for interferometry

The spatial mode is implemented by a birefringent calcite BD with length 28.165 mm and clear aperture 33 mm × 15 mm. The optical axis of each BD is cut so that vertically polarized light is directly transmitted and horizontal light undergoes a 3 mm lateral displacement into a neighboring mode which interferes with the vertical light in the same mode. Each pair of BDs forms an interferometer. Only odd (even) sites of the walker are labeled at each odd (even) step, since the probabilities of the walker appearing on the other sites are zero.

Ten BDs are placed in sequence and need to have their optical axes mutually aligned. Coalignment ensures that beams split by one BD in the sequence yield maximum interference visibility after passing through a HWP and the next BD in the sequence.

Output photons from the interferometric network are coupled into a single-mode optical fibre and subsequently detected by a single-photon APD in coincidence with the trigger photon. We characterize the quality of the experimental QW by its 1-norm distance^3^ from the simulated QW according to 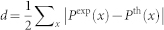. The distance increases monotonically with each step number due some lack of of relative phase control between the multiple interferometers. This limited control is due to nonplanar optical surfaces.

### Phase shifters

We introduce microscope slides into the interferometric network after first completing the alignment described above and ensuring maximum interference and small distance between simulated and empirical walker distributions. The microscope slides are inserted and aligned to recover the case of zero phase shift *ϕ* = 0 for all locations. This microscope-slide alignment corresponds to each slide being in the plane perpendicular to the beams in each path.

After achieving this alignment, each microscopic slide can be adjusted to an effective thickness in order impart a controllable PS independent from all other PSs. This effective thickness is achieved by rotating the slide out of the plane perpendicular to the beams. Tilting the slide is a viable alternative but is not as stable for the times required to gather the data.

## Author Contributions

P.X. designed the experiment, developed the theory pertinent to the experiment, analyzed the data, supervised H.Q. and B.T. on the experiment and wrote most of the paper. H.Q. and B.T. performed the experiment and collected the data.
